# Would frontal midline theta indicate cognitive changes induced by non-invasive brain stimulation? A mini review

**DOI:** 10.3389/fnhum.2023.1116890

**Published:** 2023-07-14

**Authors:** Ester Miyuki Nakamura-Palacios, Aldren Thomazini Falçoni Júnior, Quézia Silva Anders, Lucas dos Santos Pereira de Paula, Mariana Zamprogno Zottele, Christiane Furlan Ronchete, Pedro Henrique Cassaro Lirio

**Affiliations:** ^1^Department of Physiological Sciences, Federal University of Espírito Santo, Vitória, Brazil; ^2^Superior School of Sciences of the Santa Casa de Misericórdia de Vitória (EMESCAM), Vitória, Brazil; ^3^FABRA- Higher Education Center, Vitória, Brazil; ^4^Post Graduation Program in Physiological Sciences, Federal University of Espírito Santo, Vitória, Brazil

**Keywords:** frontal midline theta (FM-theta), executive functions (EFs), working memory (WM), cognitive control, non-invasive brain stimulation (NIBS), transcranial direct current stimulation (tDCS), transcranial alternate current stimulation (tACS), transcranial magnetic stimulation (TMS)

## Abstract

To the best of our knowledge, neurophysiological markers indicating changes induced by non-invasive brain stimulation (NIBS) on cognitive performance, especially one of the most investigated under these procedures, working memory (WM), are little known. Here, we will briefly introduce frontal midline theta (FM-theta) oscillation (4–8 Hz) as a possible indicator for NIBS effects on WM processing. Electrophysiological recordings of FM-theta oscillation seem to originate in the medial frontal cortex and the anterior cingulate cortex, but they may be driven more subcortically. FM-theta has been acknowledged to occur during memory and emotion processing, and it has been related to WM and sustained attention. It mainly occurs in the frontal region during a delay period, in which specific information previously shown is no longer perceived and must be manipulated to allow a later (delayed) response and observed in posterior regions during information maintenance. Most NIBS studies investigating effects on cognitive performance have used n-back tasks that mix manipulation and maintenance processes. Thus, if considering FM-theta as a potential neurophysiological indicator for NIBS effects on different WM components, adequate cognitive tasks should be considered to better address the complexity of WM processing. Future research should also evaluate the potential use of FM-theta as an index of the therapeutic effects of NIBS intervention on neuropsychiatric disorders, especially those involving the ventral medial prefrontal cortex and cognitive dysfunctions.

## Introduction

Few years ago, when investigating clinical effects of non-invasive brain stimulation (NIBS) technique applied over the dorsolateral prefrontal cortex (dlPFC) in substance use disorders, we looked for biological evidence. Using low-resolution brain electromagnetic tomography (LORETA) focused on event related potential (ERP) P3 segment, we observed that a region located more ventrally in the medial prefrontal cortex (the ventral medial prefrontal cortex, vmPFC) showed the highest electrophysiological change under drug-related cues in patients with alcohol or crack-cocaine use disorders maintaining abstinence after multiple sessions of the bilateral transcranial direct current stimulation (tDCS) over the dlPFC (Nakamura-Palacios et al., 2016). With this analysis, together with few neuroimaging data, we showed that NIBS application over the dorsal region of the prefrontal cortex (PFC) could produce changes in brain activation of the medial frontal region that were not directly target (Nakamura-Palacios et al., 2016). Following, we started to search for what could be underneath this evidence.

Most neuropsychiatric disorders are associated with important dysfunction in frontal regions, more specifically the vmPFC (Schneider and Koenigs, 2017; Hiser and Koenigs, 2018). This brain region plays a crucial role in a multitude of complex psychological functions that underlie adaptive human behavior, notably in value-based decision-making, emotion-related psychophysiology and social cognition (Schneider and Koenigs, 2017).

Brain oscillations have been suggested as biological markers in neuropsychiatric disorders (Yener and Basar, 2013), although Newson and Thiagarajan (2018) pondered that power changes within specific frequency bands may not be specific to a particular disorder. They may overlap substantially across disorders and may show a great variability within disorders (Newson and Thiagarajan, 2018).

However, frontal midline theta oscillations seems to be related to anxiety conditions and have been suggested as an instrument to evaluate symptoms of anxiety in generalized anxiety disorder as its appearance seems to be closely related to improved anxiety symptoms under treatment with anti-anxiety drugs (Suetsugi et al., 2000). It also seems to be associated with depression as the induction of frontal theta oscillations by the repetitive transcranial magnetic stimulation (rTMS) facilitates giving-up behavior with a consequent lower tendency of mental rumination, a risk factor for depressive states (Miyauchi and Kawasaki, 2021).

Thus, we wondered if NIBS induced changes in vmPFC activity, and probably a subsequent frontal theta synchronicity within cognitive networks could indicate therapeutic effects of these interventions, especially when aiming to improve cognition in neuropsychiatric disorders (Lefaucheur et al., 2020; Brunoni et al., 2021; Fregni et al., 2021).

Indeed, one major goal of NIBS, such as TMS, tDCS, or transcranial alternating current stimulation (tACS), is to promote neuroenhancement and neurorehabilitation using the potential of protocols to promote changes in cortical excitability and generate neuroplasticity in circuits underlying working memory (WM) and executive functions (EFs) (Brunoni and Vanderhasselt, 2014; Hara et al., 2021; Antal et al., 2022). However, neurophysiological markers associated with their impact on cognition and behavior are little known. In a recent systematic review and metanalysis, Mendes et al. (2022) suggested that the parietal ERP P3 amplitude could be a potential neural signature for changes induced by frontal anodal tDCS on cognitive performance, more specifically during oddball and n-back tasks.

Considering the evidence in the literature showing that theta waves consistently occur in the frontal midline during the processing of memory and emotion related to WM and EFs, we started to explore whether this electrophysiological rhythm could indicate the effects of NIBS on these cognitive processes. We first searched for articles published in the last 10 years (2012–2022) on the MEDLINE/PUBMED database, using the terms (defined by Medical Subject Headings–MeSH): “Theta Rhythm” combined (using the Boolean operator AND) with “Transcranial Direct Current Stimulation,” “Transcranial Alternating Current Stimulation,” “Transcranial Magnetic Stimulation,” “Medial Prefrontal Cortex,” resulting in 478 articles. 365 were excluded initially because of title inadequacy followed by abstracts that did not fit within the scope of our goal. The remaining 113 publications underwent full reading, of which 49 were considered relevant to be included in this brief review. Additionally, 14 articles were subsequently included to support the context.

Frontal midline theta oscillations were first named FM-theta (or Fmθ) by Ishihara and Yoshi (1972) when they were observed in juvenile delinquents performing continuous arithmetic calculations. The recordings of theta rhythms possibly originate in the medial frontal cortex (MFC) and the anterior cingulate cortex (ACC) (Cavanagh and Frank, 2014; Tollner et al., 2017), but the thalamus possibly drives them, although this requires further investigation (Mitchell et al., 2008).

Frontal midline-theta has been associated with broad cognitive functions (Cavanagh and Frank, 2014; Hsieh and Ranganath, 2014; Ishii et al., 2014; Herweg et al., 2020) and it has been suggested that it would indicate different processes depending on task requirements (Eschmann et al., 2018). According to Mitchell et al. (2008), the cognitive function of FM-theta at any particular temporal point will depend on which other structures the frontal region is interacting with in that moment, resulting in very distinct cognitive competences sharing a common level or means of computation. Additionally, Berger et al. (2019) suggested a role of gatekeeper to FM-theta, which would provide an efficient mechanism allowing or preventing remote neocortical areas to have access to PFC cognitive resources depending on cognitive demands.

The characteristics of FM-theta were detailed by Mitchell et al. (2008) and some will be depicted here. These theta oscillations are maximal in the frontal-midline regions (F3, Fz, F4) observed in electrophysiological (EEG) studies. It is mostly found around 6 Hz in sinusoidal waveforms with amplitude around 50–75 μV during behavioral tasks. It usually appears in discrete bursts for a few seconds but may range from 1 to >10 s; it tends to “wax and wane” and maybe phase- and time-locked related to behavioral tasks.

It is also essential to consider a few limitations. For instance, it seems that not all individuals display theta oscillations on EEGs signals during cognitive task performance, being of low occurrence, especially in old adults (Mitchell et al., 2008). Thus, it appears to be age-related and is most common in young adults, with its occurrence decreasing significantly after 30 years of age (Mitchell et al., 2008). However, these data were mainly based on FM-theta spontaneous induction during cognitive task performance. When using signal processing techniques and, especially, when it is evoked by events (Mitchell et al., 2008) using time-locked paradigms (Doppelmayr et al., 2000; Kieffaber et al., 2023), its occurrence is larger.

## WM and FM-theta

Here we will bring evidence proving the relationship between FM-theta and WM. Let us start by defining WM. It can be defined as a system or a process constituted by multiple components or a working system assembling distinct cognitive processes (Baddeley, 2003; Repovs and Baddeley, 2006). It contains two distinct components (Smith and Jonides, 1999) or sub-systems (Ratcliffe et al., 2022). One is a short-term storage for active maintenance, or a representational system, holding task-relevant information content for a limited period, possibly distributed in posterior regions, such as occipital/parietal or temporal areas, depending on task demands (Kawasaki et al., 2014). The other component is a set of “executive processes” associated with goal-directed behavior that operates on the content of the storage or representational system managed by the PFC and related networks (Smith and Jonides, 1999; Friedman and Robbins, 2022; Menon and D’Esposito, 2022; Ratcliffe et al., 2022).

Thus, WM processing requires the storage buffer and central EFs to be coordinated with the contribution of different brain regions with diverse functional roles. The neural mechanisms governing these processes seem to be signaled by theta and alpha waves detected in EEG recordings (Kawasaki et al., 2010). The relationship between theta and alpha oscillations was examined by Riddle et al. (2020) in a functional magnetic resonance imaging (fMRI) acquired during a retro-cued delayed WM task performance. They confirmed a model in which theta oscillations are excitatory to neural activity at frontal sites and alpha oscillations are inhibitory at posterior (parietal) sites, and they need to be balanced for WM performance (Riddle et al., 2020).

Behavioral tasks that seem to better examine the neural mechanisms underlying WM functions are those with delayed response (Funahashi, 2017). In these tasks a cued stimulus presented is no longer shown during a delay period, usually for seconds or longer. Over this time interval the information regarding the stimulus must be kept online (and manipulated or not according to the given instruction). By the end of the delay period the information needs to be retrieved from the temporary storage (maintenance) to allow the response to be given for task completion.

Patterns of enhanced FM-theta coherence, meaning increased synchronization between two cortical sites, seem specific to the delay period in delayed response tasks (Sarnthein et al., 1998). Moreover, FM-theta power seems to increase parametrically with memory load and is sustained during the delay period (Jensen and Tesche, 2002). Additionally, Sauseng et al. (2002) showed that evoked theta oscillations spread from anterior to posterior EEG recording sites when an individual retrieves information previously encoded in long-term memory system. They reverse the direction to frontal areas when information is successfully retrieved, a phenomenon they suggested reflects the transfer of information between WM and long-term memory systems.

Eschmann et al. (2018) found greater FM-theta activity (indicated by frequency power) in conditions with a high need for cognitive control. Still, it was focally activated in frontal sites in proactive (delayed matching to sample) tasks, yet broadly distributed in a reactive task (Stroop test). Ratcliffe et al. (2022), using a delayed matching to sample task and introducing a delayed procedure in one- and two-back tasks, showed focal FM-theta power increases with WM engagement during the delay interval. Decoding the WM content is driven by posterior sites, which grows with functional theta coupling to fronto-medial channels. In addition, they observed that FM-theta frequency speed (ranging from 4 to 8 Hz) dropped toward the lower end (peaking at 5 Hz) with increased WM load (one- vs. two-back tasks).

Indeed, FM-theta power seems to rise in conditions with high WM load and task difficulty, and the increase in theta activity during WM also appears to predict later long-term memory retrieval for correct responses as it has not been observed when the responses are incorrect (Eschmann et al., 2018).

Kawasaki et al. (2010), found that FM-theta was observed when information was manipulated during delay periods in auditory and visual WM tasks, but not during maintenance periods, whereas alpha wave increases in both manipulation and maintenance periods in the temporal area for the auditory WM tasks and the parietal area for visual WM tasks. Furthermore, FM-theta phase synchronization (the phase relation of theta oscillation between two different brain regions) (Fell and Axmacher, 2011) occurred only between the to-be-manipulated modality-related brain regions, such as frontoparietal synchronization for the visual modality and frontotemporal synchronization for the auditory-verbal modality (Kawasaki et al., 2014).

However, according to Berger et al. (2019) FM-theta occurs irrespectively if information is manipulated or not (retention only) during the delay period (Berger et al., 2019). According to these authors, the complexity of cognitive demand (number of items and if it is a simple retention or if mental manipulation is required) seems to define where the posterior (parietal and/or temporal) fast rhythm (gamma) will be nested into prefrontal slow waves (peak or trough expressed by FM-theta). When fast rhythms are nested in FM-theta troughs, they engage the dynamic coupling of fronto-temporo-parietal control networks, allowing the performance of a complex WM task. When gamma is nested in FM-theta peak a network decoupling seems to happen when performing a less demanding WM task.

## FM-theta, WM, and NIBS

In this section we will introduce NIBS effects on WM followed by electrophysiological evidence under these conditions. Although FM-theta has been consistently observed during many cognitive tasks, here we will focus on WM. This is because this cognitive function is one of the most studied under NIBS. Most of these studies have investigated the effects of the stimulation of the left dorsolateral prefrontal cortex (dlPFC) on the performance of n-back tasks, but, unfortunately, showing mixed results.

N-back tasks involve the continuous presentation of items, in which the participant must indicate whether an actual item presented is the same as presented one-, two-, three-, or n-times back, usually with no delay introduced, so items are presented consecutively. A clear relationship exists between this task and FM-theta spectral activity, attributed to the allocation of attentional resources related to WM (Mitchell et al., 2008). However, WM components are not well distinguished with this method.

In their meta-analysis, Brunoni and Vanderhasselt (2014) conclude that repetitive application of TMS (rTMS) over the dlPFC significantly increases accuracy and hastens response time. In contrast, tDCS improves only one parameter, the response time, with no effect on accuracy.

However, Zaehle et al. (2011) observed that anodal tDCS increases WM performance measured by a letter two-back task and amplifies oscillatory power in the theta and alpha bands in the occipito-parietal region. The cathodal tDCS, on the other hand, decreases oscillatory power in the theta and alpha bands in these posterior electrode sites and interferes with the regular repetition-related increase in WM performance. With these findings they suggested that tDCS over the left dlPFC would modulate WM performance by altering the underlying oscillatory brain activity according to its polarity (Zaehle et al., 2011).

Hoy et al. (2013) found greater theta event-related synchronization and alpha event-related desynchronization at the frontal (F3) site immediately following stimulation with a low dose of the anodal tDCS over the left dlPFC, which improved two-back task performance.

Using a delayed WM task, Jones et al. (2020) observed that anodal tDCS over the right dlPFC (F4) alternating with the right parietal cortex (P4), in a counterbalanced order, increased the phase-amplitude coupling between PFC theta oscillations and parietal gamma activity which was associated with WM training enhancement.

Differently from tDCS, a frequency-modulated brain stimulation can be implemented with TMS and tACS (Lage et al., 2016; Lefaucheur, 2019; Hosseinian et al., 2021). These techniques can be applied at similar endogenous frequencies of interest, such as theta, alpha, gamma, or at high frequencies watching for repercussions over neural oscillations.

Studies investigating the tACS, especially in theta and gamma frequencies, most over the left dlPFC, with some including parietal cortex, show inconsistent results on WM, especially in young, healthy participants or in a single session. Yet, it seems more favorable, either in one or in multiple sessions, in low performers such as old adults and patients with neurodegenerative diseases (Al Qasem et al., 2022).

Chander et al. (2016) observed that when FM-theta oscillations are phase locked by tACS applied over FPz and Pz in young subjects, the power increase of endogenous FM-theta is blocked, and WM performance measured by a two-back task is impaired. This suggests that endogenous FM-theta phase regulation is required for the stabilization and maintenance of temporal order information for correct responses in n-back tasks (Chander et al., 2016). Using similar electrode locations (FPz and Pz) but with tACS with frequency below the individual theta frequency, Vosskuhl et al. (2015) observed an increase in short-term memory capacity measured by forward digit span during the tACS, but, the WM, measured by backward digit span and three-back task, was not affected. Considering a theta-gamma coding theory, in which each gamma wave represents one memory item and each theta wave represent the list of items stored to be recall, Vosskuhl et al. (2015) suggested that lower theta frequencies would fit more gamma waves onto each theta wave.

When applied bilaterally over the dlPFC, tACS improved verbal WM accuracy in young subjects (Meiron and Lavidor, 2014), and when combined with TMS, both applied bilaterally over F3 and F4, in a complex phase-synchronized low-intensity electric and magnetic stimulation technique as developed by Hosseinian et al. (2021), a narrowband 6-Hz theta oscillations was induced and stabilized and processing of WM was enhanced.

Using TMS alone delivered in theta or alpha frequencies over the left frontal or left parietal region, matching or not to the task-driven theta or alpha oscillations, seen by neuroimaging analysis during a retro-cued delayed WM task, Riddle et al. (2020) demonstrated a causal role for theta and alpha neural oscillations in, respectively, prioritization and suppression of WM representations. They suggested that an optimal excitatory-inhibitory (or theta-alpha) balance at the fronto-parietal network is required to manage a successful WM performance (Riddle et al., 2020).

Employing TMS at high frequency to frontal (Fz), temporal (TP7) or parietal (Pz) areas paired with EEG during an auditory delayed WM task, Miyauchi et al. (2016) explored the directionality of the theta phase synchronization between frontal and sensory areas. They found that TMS increased theta phase synchronization when delivered over sensory areas, but not when delivered over frontal area, suggesting that theta phase synchrony induced by TMS during WM processing was bottom-up directed.

Berger et al. (2019) also delivered a fast (50 Hz) triple-pulse of TMS over the right temporo-parietal site during the through of the FM-theta cycle and found that a delayed task performance was disrupted. This result proved a causal relationship between the nesting of posterior (temporo-parietal) gamma bursts into specific FM-theta phases. They suggested that task-relevant temporo-parietal neural activity must be pulsed and synchronized to FM-theta waves to guide an efficient WM performance (Berger et al., 2019).

## Neurochemical and molecular mechanisms underlying FM-theta and NIBS

So far, evidence is growing that NIBS may induce FM-theta oscillations related to WM processing. To that end, they likely share biochemical and molecular mechanisms.

Few neurochemistry studies have associated the variation in FM-theta with variation in monoamine (dopamine, noradrenaline, and serotonin) systems. It seems that not only their levels but the balance between them is important in the control of this frontal rhythm (Mitchell et al., 2008).

Anodal tDCS and TMS enhance excitatory synaptic transmissions possibly by facilitating cortical glutamate transmission and suppressing gamma-aminobutyric acid transmission. They also modulate positively or negatively the activities of dopamine, serotonin, and acetylcholine transmissions, events that may change the balance between excitatory and inhibitory inputs (Chervyakov et al., 2015; Yamada and Sumiyoshi, 2021).

The molecular basis of the synchronicity of theta oscillation between the medial prefrontal cortex and amygdala and hippocampus in conditioning responses associated with fear and reward in rodents, seems to be associated with the expression of receptors and proteins fundamental to the process of brain plasticity, such as NMDA (N-methyl D-Aspartate) receptors, dopamine receptors, c-FOS and CREB (cyclic-AMP response element binding protein) (Bocchio et al., 2017; Anders et al., 2018).

These molecular mechanisms also mediate cortical changes induced by NIBS (Pelletier and Cicchetti, 2014; Chervyakov et al., 2015; Yamada and Sumiyoshi, 2021). Animal studies have shown that tDCS enhances the expression of NMDA and BDNF (Brain-Derived Neurotrophic Factor) and involves AMPA (α-amino-3-hydroxy-5-methyl-4-isoxazolepropionic acid) receptor modulation and GAP-43 (cortical growth-associated protein) expression, possibly related to improving cognitive performance (Wu et al., 2017; de Souza Custodio et al., 2018; Martins et al., 2019; Nakamura-Palacios et al., 2021). However, Serrano et al. (2022) observed a reduction of serum BDNF which was correlated with the improvement of WM and other cognitive tasks induced by home-based anodal tDCS in patients with fibromyalgia. Regarding TMS, long-lasting therapeutic effects seem to be related to LTP (long-term potentiation) and LTD (long-term depression) involving post-synaptic NMDA receptors, changing BDNF production in stimulated and remote brain regions, but increased, unchanged, or decreased in the serum and cerebrospinal fluid (Chervyakov et al., 2015; Lefaucheur, 2019). There is also evidence that tACS may also induce plasticity mediated by NMDA receptors in the motor cortex, because the after-effects of 20 Hz tACS was suppressed by a NMDA antagonist (Wischnewski et al., 2019, 2023) and may potentially induce BDNF changes (He et al., 2023).

## Conclusion

Bearing in mind experimental limitations and limited evidence, FM-theta could be a potential electrophysiological indicator for NIBS effects on distinct WM components. Furthermore, FM-theta oscillations could also indicate the potential therapeutical effects of NIBS on neuropsychiatric disorders associated with vmPFC and cognitive dysfunctions. Evidently, this hypothesis needs to be specifically investigated in the future.

## Author contributions

EN-P conceived the original idea, directed the discussion of references included in the text, assembled the parts worked by the other authors, and wrote the main text. AF schematized the search keys in the literature with all the other authors and drafted the introduction. QA, LP, MZ, CR, and PL worked and drafted parts of the text included in the main text. All authors discussed the content and contributed to the final manuscript.
